# Single-cell molecular communications and transcriptional regulatory dynamics of T cell immunotherapy in bladder cancer

**DOI:** 10.1016/j.gendis.2025.101760

**Published:** 2025-07-03

**Authors:** Limin Liu, Xuan Wan, Su Liu, Chenjie Yu, Hongman Xue, Jiayi Wang, Zhu Li, Kai Liu, Chun Chen, Jiajian Wang

**Affiliations:** a Gynecology Department, Shenzhen Maternity and Child Healthcare Hospital, Women and Children's Medical Center, Southern Medical University, Shenzhen, Guangdong 518028, China; b Chronic Airways Diseases Laboratory, Department of Respiratory and Critical Care Medicine, Nanfang Hospital, Southern Medical University, Guangzhou, Guangdong 510515, China; c Pediatric Hematology Laboratory, Division of Hematology/Oncology, Department of Pediatrics, The Seventh Affiliated Hospital of Sun Yat-Sen University, Shenzhen, Guangdong 518107, China; d First Affiliated Hospital of Anhui Medical University, Hefei, Anhui 230022, China; e First School of Clinical Medicine, Anhui Medical University, Hefei, Anhui 230032, China; f School of Basic Medical Sciences, Anhui Medical University, Hefei, Anhui 230032, China; g Department of Dermatology, The Seventh Affiliated Hospital of Sun Yat-sen University, Shenzhen, Guangdong 518107, China; h Scientific Research Center, The Seventh Affiliated Hospital, Sun Yat-sen University, Shenzhen, Guangdong 518107, China

Immunotherapy shows promise in treating various cancers, yet solid tumors like bladder cancer often exhibit immune resistance. This study explored T cell dynamics in bladder cancer, focusing on how different T cell subtypes communicate and respond to therapy. We analyzed the transcriptional activities and intercellular signaling of CD4^+^ and CD8^+^ T cells, aiming to optimize therapeutic strategies and enhance immune response efficacy.

Transcriptional activity in T cells reflects a regulatory response to the tumor microenvironment, offering clinical insights into immune surveillance and therapeutic strategies. We analyzed specific transcription factors across various T cell subtypes under different treatment regimens to uncover differences in transcriptional regulation [Fig. 1A–C (tumor samples: A, B; normal tissue samples: C); Fig. S1A–H (tumor samples: A, B, E, F; normal tissue samples: C, D, G, H) and Table S2–S13]. Notably, CD4PROLIF and CD8ENTPD1 T cells displayed active regulons, underscoring the dynamic communication within and between cells. The transcription factors active in these cells varied by treatment, revealing a complex interaction landscape influenced by the therapy administered. In the absence of systemic therapy, CD4^+^ T cells predominantly expressed the CD4IL2RAHI and CD4MITO phenotypes, whereas CD8^+^ T cells were mainly CD8ENTPD1 and CD8PROLIF. Under anti-PD-L1 treatment, CD4ACTIVATED, CD4GZMB, CD4IL2RAHI, and CD4MITO, along with CD8ENTPD1, CD8HSP, and CD8PROLIF, were highly active, illustrating treatment-specific transcriptional activation linked to tumor killing. In contrast, chemotherapy predominantly activated CD8^+^ immune cell subtypes.

**Figure 1:**
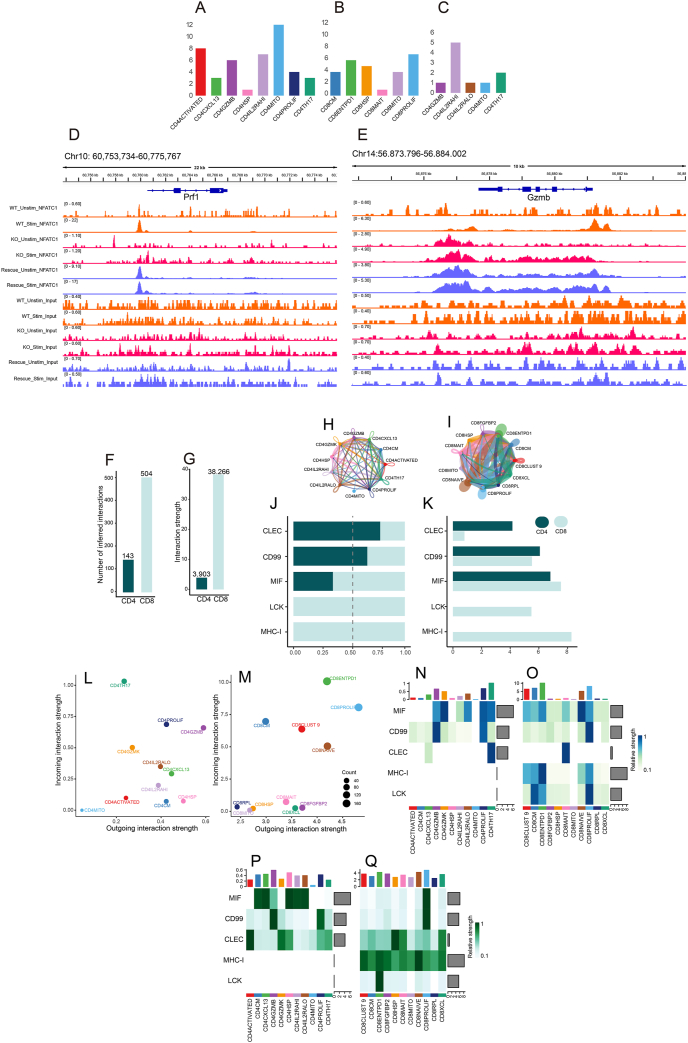
Transcriptional dynamics and intercellular communication in CD4/CD8 T cells during anti-PD-L1 immunotherapy. **(A**–**C)** Frequency statistics of specific transcription factors for different cell subtypes in the anti-PD-L1 therapy group. A and B represent the frequency of transcription factors in tumor samples under anti-PD-L1 immunotherapy, while C represents the frequency of transcription factors in normal samples under anti-PD-L1 immunotherapy. The horizontal coordinates indicate the different cell subtypes and the vertical coordinates indicate the frequency of specific transcription factors. **(D, E)** NFATC1 directly binds to the promoters and enhancers of the cytotoxic genes *PRF1* and *GZMB* in a stimulation-dependent and genotype-specific manner. IGV genome browser views of NFATC1 ChIP-seq data and corresponding input control tracks at the *PRF1* (chr10:60,753,734–60,775,767) and *GZMB* (chr14:56,873,796–56,884,002) loci in mouse CD8^+^ T cells (mm9) are shown. Strong NFATC1 binding peaks were detected at the *PRF1* promoter and the *GZMB* promoter and upstream enhancer elements exclusively in wild-type (WT) and CA-RIT–NFATC1-rescued cells upon PMA/ionomycin stimulation. No binding was observed in NFATC1 knockout (KO) samples under either condition or in unstimulated WT and Rescue cells. The input tracks displayed uniformly low background signals across all conditions, validating the specificity of the ChIP. A total of 12 tracks are shown for each locus: the top six correspond to NFATC1 ChIP-seq signals, and the bottom six are input controls. The sample metadata are as follows: WT unstimulated, GSM1570756 (ChIP) and GSM1570750 (input); WT stimulated, GSM1570759 (ChIP) and GSM1570753 (input); KO unstimulated, GSM1570757 (ChIP) and GSM1570751 (input); KO stimulated, GSM1570760 (ChIP) and GSM1570754 (input); Rescue unstimulated, GSM1570758 (ChIP) and GSM1570752 (input); Rescue stimulated, GSM1570761 (ChIP) and GSM1570755 (input). ChIP-seq color coding is consistent across genotypes, including orange (WT), magenta (KO), and blue (Rescue). These results provide direct *in vivo* evidence that NFATC1 transcriptionally regulates *PRF1* and *GZMB* through physical DNA binding but only in the presence of functional NFATC1 protein and sufficient activation signaling, thereby supporting its role as a key regulator of cytotoxic gene programs in CD8^+^ T cells. **(F–I)** Number of interactions, strength of interactions and interactions between different subtypes of T cells in the anti-PD-L1 therapy group. F and G represent the number and intensity of interactions among CD4^+^/CD8^+^ T cells under anti-PD-L1 therapy, while H and I depict the interaction network among CD4^+^/CD8^+^ T cells under anti-PD-L1 therapy. Different colors represent different cell subtypes, and the thickness of the lines indicates the strength of the interactions. **(J, K)** Incoming-outgoing interaction correlated signaling pathways and their relative proportions in the anti-PD-L1 therapy group. The vertical coordinates are abbreviated for different signaling pathways; the horizontal coordinates of J are the relative information flow, and the horizontal coordinates of K are the information flow. **(L, M)** The intensity of incoming–outgoing interactions in the anti-PD-L1 therapy group. The horizontal coordinate represents the outgoing interaction intensity, and the vertical coordinate represents the incoming interaction intensity. The different colors of the circles represent different T cell subtypes, and the cell subtypes to which the circles belong are marked with the corresponding colors. The circle size indicates the count size. **(N**–**Q)** Distribution of incoming–outgoing interaction correlated signaling pathways and their cell subtypes in the anti-PD-L1 therapy group. N represents the incoming signaling patterns of CD4^+^ T cells under anti-PD-L1 therapy, while O represents the incoming signaling patterns of CD8^+^ T cells under anti-PD-L1 therapy. P indicates the outgoing signaling patterns of CD4^+^ T cells during anti-PD-L1 therapy, and Q indicates the outgoing signaling patterns of CD8^+^ T cells during anti-PD-L1 therapy. The vertical coordinates of the heat map indicate the signaling pathways, and the horizontal coordinates indicate the different cell subtypes. The colors of heat map indicate the relative strength of different pathways in different subtypes of cells. The top of the heat map indicates the relative strengths accounted for by different subtypes of cells, and the right side of the heat map indicates the strengths accounted for by different pathways.

Several transcription factors specific to CD4^+^ T cells involved in cytotoxicity were similar to those specific to certain CD8^+^ T cells (Fig. S1I, J and Table S2–S13). In the presence of anti-PDL1 treatment, we found that these transcription factors ZN341 (zinc finger protein 341), SRBP2, WT1, FLI1, KLF1, and ZN281 were all distributed in multiple cell subtypes. The transcription factors for the chemotherapy group included VEZF1 (vascular endothelial zinc finger 1), PATZ1 (POZ/BTB and AT hook-containing zinc finger 1), NFAC1, and KLF16 (Kruppel-like factor 16), and those specific for the no systemic therapy group included WT1, STAT1, E2F7, KLF6, ZN148 (zinc finger protein 148), ZBT17 (zinc finger and BTB domain containing 17), KLF12 (Kruppel-like factor 12), TBX1, and NFAC1. We found that WT1-specific T cell receptors and KLF transcription factor family members were consistently enriched across treatment conditions, enhancing T cell effector function through calcium-dependent activation of NFATC1, a key downstream transcription factor in TCR signaling.^1^^,^^2^ To determine whether NFATC1 regulates cytotoxic genes, we analyzed ChIP-seq and RNA-seq data from CD8^+^ T cells under wild-type (WT), nuclear factor of activated T cells1 (NFATC1) knockout, and CA-RIT–rescued conditions (Fig. 1D, E and Table S14).^3^ NFATC1 showed strong binding to the *PRF1* promoter and *GZMB* enhancers in WT and rescued cells but not in knockouts. Reconstitution of NFATC1 led to significant *PRF1* up-regulation (17.3 × , *P* = 1.4 × 10^−5^), confirming its direct transcriptional role. These findings demonstrate NFATC1 as a direct transcriptional activator of *PRF1* with likely modulation of *GZMB*, highlighting the treatment-responsive transcriptional dynamics shaped by the tumor microenvironment.

The tumor immune microenvironment is characterized by the crucial role of synergistic interactions among immune cells, particularly CD4 and CD8 T cells, which are integral to maintaining environmental stability despite variations in transcriptional activity. Analysis of the interacting cell types showed a positive correlation between the number and intensity of cellular communications (Fig. 1F–I; Fig. S2). The major cell populations involved varied considerably across the different treatment states, with CD8^+^ T cells exhibiting stronger effects than CD4^+^ T cells overall (interaction strength for CD4^+^/CD8^+^ T cells: 5619/26,169 with chemotherapy, 3903/38,266 with anti-PD-L1 therapy, and 2043/11,566 with no systemic therapy; Fig. 1 F–I; Fig. S2).

Observing the flow of information among T cells within the tumor microenvironment can reveal the overall involvement of immune cells in pathways, swiftly identifying crucial signaling pathways. Under chemotherapy, CD4^+^ T cells activated the CLEC and ITGB2 pathways, while CD8^+^ T cells activated the LCK and MHC-I pathways, with an overlap observed in the MIF and CD99 pathways (Fig. 1J, K; Fig. S3). Notably, anti-PD-L1 treatment deactivated pathways such as ITGB2, CCL, CD70, and SPP1 compared to other regimens. Following anti-PD-L1 treatment, CD8^+^ T cells additionally activated the CLEC and CD99 pathways along with their usual LCK and MHC-I pathways. Conversely, CD4^+^ T cells deactivated the ITGB2 pathway. Without systemic treatment, CD4^+^ T cells maintained the primary CLEC, CCL, MIF, and CD99 pathways. In contrast, CD8^+^ T cells under chemotherapy and anti-PD-L1 blockade maintained different sets of pathways: CD70 and SPP1 for chemotherapy, and MIF, CD99, LCK, and MHC-I for anti-PD-L1 treatment.

CD4^+^ T cells differed considerably between the no systemic therapy group and the treated group (Fig. 1L, N, P; Figs. S2C, S2D, S2G, S2H; Fig. S4): (1) CD4GZMK, CD4GZMB, and CD4CXCL13 T cells were essentially unchanged in both groups, except for a smaller attenuation in the anti-PD-L1 treatment group, with some reciprocal intensity. (2) Stronger CD4ACTIVATED, CD4IL2RALO, and CD4IL2RAHI T cell interactions were found in the treated group; the CD4PROLIF T cell intensity was greater in the no systemic therapy group than in the treated group; and the intensity of CD4TH17 T cells was greater in the no systemic therapy group than in the presence of anti-PD-L1 therapy. (3) We also found that CD4HSP, CD4CD, and CD4MITO T cells were relatively inactive across treatment states and showed only a low level of reciprocal relationships and intensity.

CD8^+^ T cells exhibited a generally greater number and intensity of interactions compared to CD4^+^ T cells across both the no systemic therapy group and the treated group, with notable subtype-specific differences (Fig. 1M, O, Q; Figs. S2C, S2D, S2G, S2H; Fig. S4). CD8ENTPD1, CD8CM, and CD8MAIT cells remained consistently active across all treatment states. CD8NAIVE cells showed increased activity in the treated group but were less active following chemotherapy. CD8XCL cells exhibited reduced activity in the anti-PD-L1 group compared to both the chemotherapy and untreated groups, while CD8CLUST9 cells were active in the anti-PD-L1 and untreated groups but not in the chemotherapy group. In contrast, subtypes such as CD8HSP, CD8RPL, and CD8MITO were consistently inactive, indicating inert communication profiles.

At the single-cell level, we further delineated communication differences between CD4^+^ and CD8^+^ T cells, revealing potential synergistic interactions (Fig. 1L–Q; Fig. S4 and S5). Notably, CD4PROLIF, CD4GZMK, and CD4GZMB cells mirrored the signaling profiles of CD8CLUST9, CD8PROLIF, CD8CM, and CD8NAIVE cells before and after anti-PD-L1 therapy, suggesting functional convergence during immunotherapy. CD4TH17 and CD8ENTPD1 cells exhibited stable signaling intensities across treatments, with CD4TH17 cells primarily receiving signals and CD8ENTPD1 cells emitting signals, which was especially pronounced in the chemotherapy group. This stability implies that their core communication behaviors are treatment-independent. However, their signaling pathways remain largely distinct, overlapping primarily in the MIF pathway, highlighting MIF as a potential target in immunotherapeutic strategies.

Taken together, our findings underscore the coordinated yet distinct roles of CD4^+^ and CD8^+^ T cells in the antitumor immune response under various treatment regimens. This study provides the first systematic comparison of their cellular communication patterns, revealing consistent entry and exit signaling behaviors. These insights deepen our understanding of their functional complementarity and establish a theoretical framework for refining T cell-based immunotherapies. The observed signaling coordination suggests a synergistic response to immunotherapy, offering valuable guidance for developing more precise and personalized therapeutic strategies.^4^

## CRediT authorship contribution statement

**Limin Liu:** Validation, Resources. **Xuan Wan:** Validation, Resources. **Su Liu:** Conceptualization, Investigation. **Chenjie Yu:** Validation, Resources. **Hongman Xue:** Investigation. **Jiayi Wang:** Methodology, Software, Data curation. **Zhu Li:** Supervision, Investigation, Resources. **Kai Liu:** Software, Data curation, Formal analysis. **Chun Chen:** Investigation. **Jiajian Wang:** Writing – review & editing, Visualization, Supervision, Resources, Methodology, Funding acquisition, Data curation, Writing – original draft, Validation, Software, Project administration, Investigation, Formal analysis, Conceptualization.

## Funding

This work was supported by the 10.13039/501100012151Sanming Project of Medicine in Shenzhen, China (No. SZSM202011004, SZSM202211032); the 10.13039/501100001809National Natural Science Foundation of China (NSFC, Grant No. 82370164); the 10.13039/501100010877Science, Technology and Innovation Commission of Shenzhen Municipality, China (No. JCYJ20220530155006014, JCYJ20190809160609727, JCYJ20210324123004011, JCYJ20180307150419435).

## Conflict of interests

The authors declare no conflict of interests.
