# Supplementary material for: Single-cell molecular communications and transcriptional regulatory dynamics of T cell immunotherapy in bladder cancer
Source: Genes Dis. 2025 Jul 3;13(1):101760. doi: 10.1016/j.gendis.2025.101760 (PMC12624647; doi:10.1016/j.gendis.2025.101760)
Supplement: Multimedia component 2 [file mmc2.docx]

**Supplementary Material**

Introduction, Materials and Methods, Supplementary Results, Discussion and Supplementary Figures.

**Single-Cell Molecular Communications and Transcriptional Regulatory Dynamics of T Cell Immunotherapy in Bladder Cancer**

Limin Liu ^a,†^, Xuan Wan ^b,†^, Su Liu ^c,†^ , Chenjie Yu ^a^, Hongman Xue ^c^, Jiayi Wang ^d^, Zhu Li ^e^, Kai Liu ^a, *^, Chun Chen ^c,*^, Jiajian Wang ^c, f, *^

a Gynecology Department, Affiliated Shenzhen Maternity & Child Healthcare Hospital, Southern Medical University, Shenzhen 518028, China

b Chronic Airways Diseases Laboratory, Department of Respiratory and Critical Care Medicine, Nanfang Hospital, Southern Medical University, Guangzhou 510515, China

c Pediatric Hematology Laboratory, Division of Hematology/Oncology, Department of Pediatrics, The Seventh Affiliated Hospital of Sun Yat-Sen University, Shenzhen 518107, China

d First Affiliated Hospital of Anhui Medical university, Hefei 230022, China; First School of Clinical Medicine, Anhui Medical University, Hefei 230032, China; School of Basic Medical Sciences, Anhui Medical University, Hefei 230032 China

e Department of Dermatology, the Seventh affiliated hospital of Sun Yat-sen University, Shenzhen 518107, China.

f Scientific Research Center, The Seventh Affiliated Hospital, Sun Yat-sen University, Shenzhen, 518107, Guangdong, China

† These authors contributed equally as co-first authors

* Corresponding author

E-mail addresses: [121857345@qq.com](mailto:121857345@qq.com) (K.L.), [chenchun@sysush.com](mailto:chenchun@sysush.com) (C.C.), [jiajianwang2019@gmail.com](mailto:jiajianwang2019@gmail.com) (J.J.W. )

**Abstract**

Understanding the complex interactions between T cell subtypes during PD-1 immunotherapy for bladder cancer remains a significant challenge. The specific interrelationships between these cells and their unique transcriptional regulatory activities are crucial for maintaining tumor cell homeostasis, yet the detailed mechanisms are still not fully understood. By employing network analysis and pattern recognition techniques, we explored the communication patterns among various immune cell subtypes. Utilizing the Integrated Cell-type-specific Regulon Inference Server (IRIS3), we monitored gene regulatory dynamics across different T cells, revealing distinct communication strengths. Notably, CD4GZMK and CD4GZMB T cells demonstrated communication strengths similar to those of CD8CLUST9, CD8PROLIF, CD8CM, and CD8NAIVE T cells. However, subtypes like CD4TH17 and CD8ENTPD1 showed non-overlapping pathways, suggesting unique roles in tumor recognition and clearance. Treatment-specific responses were also observed; CD4+ T cells predominantly engaged in MIF-based pathways during anti-PD-L1 therapy, contrasting with CD8+ T cells that relied on anti-MHC class II antibodies for tumor killing. Under chemotherapy, CD4+ T cells were involved in activating ITGB2 and CD99 pathways crucial for leukocyte adhesion and migration, while CD8+ T cells activated the LCK pathway, essential for downstream T cell activation. The similarities in transcription factors such as the KLF family, WT1, and NFAC1 between cytotoxic CD4+ and CD8+ T cells underline potential targets for optimizing immunotherapeutic strategies. This comprehensive analysis contributes to a better understanding of the interactions among T cell subtypes in various treatment settings, aiding the development of more effective immunotherapy approaches for bladder cancer.

**Key words:** bladder cancer, CD4+/CD8+ T cells, immunotherapy, single-cell regulatory network, T cell subtype interactions, cell-type-specific regulons (CTSRs), single-cell transcriptome

**1.Introduction**

Immunotherapies for tumors currently in clinical use, such as immune checkpoint blockade (*1, 2*), vaccines (*3-6*), T cell therapy (*7, 8*), and chimeric antigen receptor (CAR) T cell therapy (*9, 10*), are only therapeutically effective in producing good long-term patient responses or even a cure in specific patient groups and tumor types (such as hematological tumors and skin tumors). However, a large body of patient groups including those with solid tumors, fail to achieve long-term responses to immunotherapeutic approaches. The functions of immune cells play a crucial role in understanding the effects of immunotherapy and optimizing treatment protocols.

Bladder cancer, which is characterized by a high propensity for metastasis and immune tolerance, has also become an increasingly prominent public health problem (*11, 12*). Both cytotoxic CD4+ T cells and CD8+ T cells are effector immune cells in bladder cancer (*13*). Sustained antigenic stimulation in the tumor microenvironment causes T cell exhaustion, and bladder cancer in particular, with a high rate of somatic mutations, produces many tumor antigens, allowing for heterogeneous and complex phenotypic changes in T cells during immunotherapy (*14, 15*). These molecular features require that we need to thoroughly grasp the activity pattern of these T cells in order to sort out the activity among them. Currently, exhausted CD8+ T cells are the main T cell subtype targeted by immunotherapy-induced antitumor immune mechanisms (*16*). However, the efficacy of the use of immune checkpoint inhibitors (ICIs) alone is only 20% (*17, 18*). Another subtype of T cells, CD4+ T cells, is prone to depletion and may contribute to ICI in a variety of solid tumors, such as bladder cancer reactivation of antitumor immune responses after treatment (*13, 19, 20*). Oh et al. identified heterogeneous states for CD4+ T cells during bladder cancer immunotherapy, in which Tregs differentiated on the basis of IL2RA (interleukin 2 receptor subunit alpha) and immune check proteins, whereas cytotoxic CD4+ T cells differentiated on the basis of cytotoxic effector molecules such as GZMB (granzyme B) and GZMK (granzyme K) (*13*). Thus, the balance between CD4+ T cells and cytotoxic CD4+ T cells is also important for tumor immunotherapy (*13*). Zheng et al. found that the follicular helper T cell (T_FH_)/T helper 1 (T_H1_) dual-function T cells of the CD4+ T cells may originate from T_FH_ cells, which as a potentially tumor-reactive T cell population associated with the tumor mutation burden (*21*). Interestingly, some CD4+ and CD8+ T cells have cellular subtypes with similar transcriptional expression, such as CD4_GZMB_ and CD8_ENTPD1_ T cells, and CD4_GZMK_ and both CD8_CM_ and CD8_NAIVE_ T cells (*13*). Cytotoxic CD8+ T cells of different subtypes occupy a large proportion of many solid tumors, and this cell type is the most prominent cell type in immunotherapy (*22-25*). Zheng et al. suggested that the depleted state of CD8+ T cells is a population of potentially tumor-reactive T cells that includes both effector memory T cells and tissue-resident memory T cells, and that these states are associated with a variety of immune cells (e.g., natural killer cells, type 17 CD8+ T cells (Tc17 cells) and CD8+ Treg cells) in state transition and communication (*21*). Throughout the tumor microenvironment, CD8+ T cells are sparsely infiltrated in altered-immunosuppressed tumors, usually at the periphery of the tumor, with the presence of immunosuppressive cells. In altered-excluded immune tumors, the tumor microenvironment is depleted of CD8+ T cells and the main features of this type are dense stroma and hypoxia, which make it difficult for immune cells to survive (*26, 27*). T cells are faced with multiple layers of difficult “isolation” by tumor cells. Understanding the interactions or synergistic patterns among immune cells and readjusting the balance and ratio of virulent T cells to immune cells are essential to break the isolation of tumors.

During immunotherapy treatments, tumor-reactive T cells present in some patients are able to proliferate, infiltrate the tumor, and eliminate cancer cells. Is the response of these tumor-reactive T cells in immunotherapy-resistant or non-responsive patients optimally activated or amplified? How do the different subtypes of tumor-reactive T cells work together as anti-tumor effectors? The extent to which these different T cell subtypes contribute to immunotherapeutic effects are not fully understood. In order to address these questions, the signaling connections among cells must be identified and the patterns connections analyzed (*28*). Our study used single-cell CD4+ and CD8+ T cell RNA sequencing datasets before and after immunotherapy to quantitatively analyze and infer intercellular communication networks as well as characterizing the differences of communication among different immune cell subtypes. By doing so, we assessed which T cell subtypes were more active under different treatment strategies and which T cell subtypes contributed the most to immune therapy. Our main aim was to classify immune T cell intercommunication signals in bladder cancer and find key transduction signaling pathways in addition to delineating conserved and specific signaling pathways. Finally, we next analyzed the specific regulons within bladder cancer immune T cells to further explore how the transduction signals communicated externally with other cells and how the intracellular regulons functioned in a coordinated manner (*29*). The coordination and unification of these intracellular and external signals will facilitate further exploration of the manner in which bladder cancer immune cells work in concert with each other, that could potentially have important implications for developing both immunotherapies and clinical protocols.

**2.Materials and Methods**

**2.1 Generation and Construction of Cell Communication Network Objects**

The createCellChat function utilizes an expression matrix of single-cell transcriptomes and cell annotation data to construct an object format compliant with CellChat (*5*). CellChat infers ligand-receptor pairs and their quantities using the "trimean" averaging method by default for each cell subpopulation. The computeAveExpr function allows for the examination of genes of interest, such as computeAveExpr(cellchat, features = c("PD1", "PDL1"), type = "truncatedMean", trim = 0.1). Subsequently, the computeCommunProb function is employed to analyze the probability of cell communication: cellchat <- computeCommunProb(cellchat). Communication probabilities are further analyzed using the computeCommunProbPathway function: cellchat <- computeCommunProbPathway(cellchat). The data for this analysis, including immune cell single-cell datasets, were sourced from the GSE149652 dataset in the GEO database. The identification of cell type marker genes primarily follows the classifications described by D. Y. Oh et al (**Supplementary Tables 1**) (*13*).

**2.2 Analysis of the Role and Heterogeneity of the Cellular Communication Networks**

Initially, the communication network is weighted to identify dominant senders, receivers, mediators, and influencers using cellchat <- netAnalysis_computeCentrality(cellchat, slot.name = "netP") and netAnalysis_signalingRole_network(cellchat, signaling = pathways.show, width = 8, height = 2.5, font.size = 10). The coordinated and unified patterns of the network's input and output signaling pathways are analyzed using selectK(cellchat, pattern = "outgoing") and selectK(cellchat, pattern = "incoming"). These allow for the identification of communication patterns using the identifyCommunicationPatterns function, and visualization of specific pathways through netAnalysis_river or netAnalysis_dot functions. Communication pathway heterogeneity is further investigated using computeNetSimilarity, netEmbedding, netClustering, and netVisual_embedding, grouping and visualizing pathways effectively.

**2.3 Comparative Analysis of Various Cellular Communication Networks**

Comparative analysis begins by examining the number and intensity of interactions with the compareInteractions function, which helps to distinguish between different single-cell datasets collected under varying conditions. The netVisual_diffInteraction and netVisual_heatmap functions visualize these differences in interaction number and intensity. Outgoing and incoming interaction strengths are compared two-dimensionally with netAnalysis_signalingRole_scatter, identifying cell populations where signal transmission or reception significantly varies across datasets.

**2.4 Inference of Cell Type-Specific Regulons**

IRIS3 was chosen for analyzing cell type-specific regulons due to its integration of tools like DrImpute, Seurat, QUBIC2, DMINDA2, MEME, and Slingshot (*29-35*). IRIS3's process settings include DrImpute’s default "no", Seurat’s 10 principal components, 5000 highly variable features, and a clustering resolution of 0.8; QUBIC2's bicluster overlap rate of 0.7, a maximum of 500 biclusters, minimum cell number of 20, discretization level of 0.06, and consistency level of 1.0; and DMINDA2 and MEME's promoter region analysis set to 1,000 bases upstream.

**3.Supplementary Results**

**3.1 Molecular Characterization of the Intracellular Transcriptional Regulatory Network of CD8+ T and CD4+ T Cell Subtypes**

To explore the transcriptional regulatory networks among CD8+ and CD4+ T cells in bladder cancer, we analyzed single-cell transcriptomic data from seven patients with muscle-invasive bladder cancer. This cohort included two patients without systemic therapy, one receiving chemotherapy with gemcitabine and carboplatin, and four undergoing anti-PD-L1 therapy. The dataset comprised 8,833 tumor-derived CD8+ T cells, 16,995 CD4+ T cells, 1,929 non-malignant tissue-derived, and 2,847 non-malignant tissue-infiltrate CD4+ T cells (*13*). Utilizing the IRIS3 single-cell RNA-Seq process, we enhanced our analysis of transcription factors and their target genes. This approach integrated tools such as DrImpute (*34*), scran (*36*), Seurat (*33*), QUBIC2 (*32*), DMINDA2.0 (*31*), and MEME (*37*) to identify and delineate the regulons and cell type-specific transcriptional activities within these subpopulations, providing a refined understanding of the differential regulatory networks influencing bladder cancer T cells.

In order to capture the differences more precisely in transcription factors specific to different treatment contexts, we focused on transcription factors specific to the different treatments. By analyzing the immune T cell regulons of the different treatment regimens, we found that the immune cell regulatory network (particularly specific transcription factor activity) was less active in the normal tissue samples than in the tumor samples (**Fig. 1 A-C (tumor samples: A and B; normal tissue samples: C), Fig. S1 A-H (tumor samples: A, B, E, and F; normal tissue samples: C, D, G and H), Supplementary Tables 2–13**). Overlapping factors in the chemotherapy and no systemic therapy groups of tumor samples included TBX1 (T-box transcription factor 1), NFAC1 (nuclear factor ff activated T cells 1), and E2F6 (E2F transcription factor 6) (**Fig. S1 I and J, Supplementary Table 13**). TBX1 exerts oncogenic effects by inhibiting the activity of the PI3K/AKT and MAPK/ERK pathways (*38*). Potentially, E2F6 is involved in the mitogenic activity of centromere protein U (CENPU), which in turn promotes cell proliferation (*39*).

There were overlapping factors in the anti-PDL1 and non-systematically treated groups including WT1 (Wilms tumor transcription factor 1), STAT1 (signal transducer and activator of transcription 1), FOXJ3 (forkhead box J3), and SRBP2 (sterol regulatory element binding transcription factor 2), transcription factors that are sensitive to extracellular factors (**Fig. S1 I and J, Supplementary Table 13**). WT1-specific T cell receptors improve the function of anti-tumor T cells (*40*). STAT1, which can be rapidly activated by a large number of cytokines, including interferons and interleukins, acts to stimulate apoptosis and inhibit tumor growth (*41, 42*). The miR-27a/FOXJ3 axis plays a central pivotal role in the regulation of mitochondrial homeostasis (*43*).

Overlapping factors in the chemotherapy and anti-PDL1 groups included ZF64A (ZFP64 sinc finger protein), FLI1 (leukemia integration 1 transcription factor), E2F1 (retinoblastoma-binding protein 3 (E2F-like)), ZN281 (zinc finger protein 281), KLF3 (basic Kruppel-like factor), and SALL4 (Spalt-like transcription factor 4) (**Fig. S1 I and J, Supplementary Table 13**). These transcription factors play a key role in both the maintenance of the immune microenvironment and immune cell transfer. FLI1 protects the transcriptional landscape of developing CD8+ T cells from ETS-RUNX-driven Teff cell over-differentiation (*44*). Furthermore, genetic deletion of FLI1 improved TEFF differentiation and protective immunity against infection and cancer (*44*). E2F1 may be involved in the formation of a lipid-rich environment in the tumor microenvironment (*45*). KLF3 was positively associated with the level of tumor immune infiltration (*46*). Interestingly, while none of the normal tissue samples had overlapping transcription factors, the bladder cancer samples had varying degrees of overlapping samples, suggesting that similar regulatory networks were present in bladder cancer tissue under different treatment regimens.


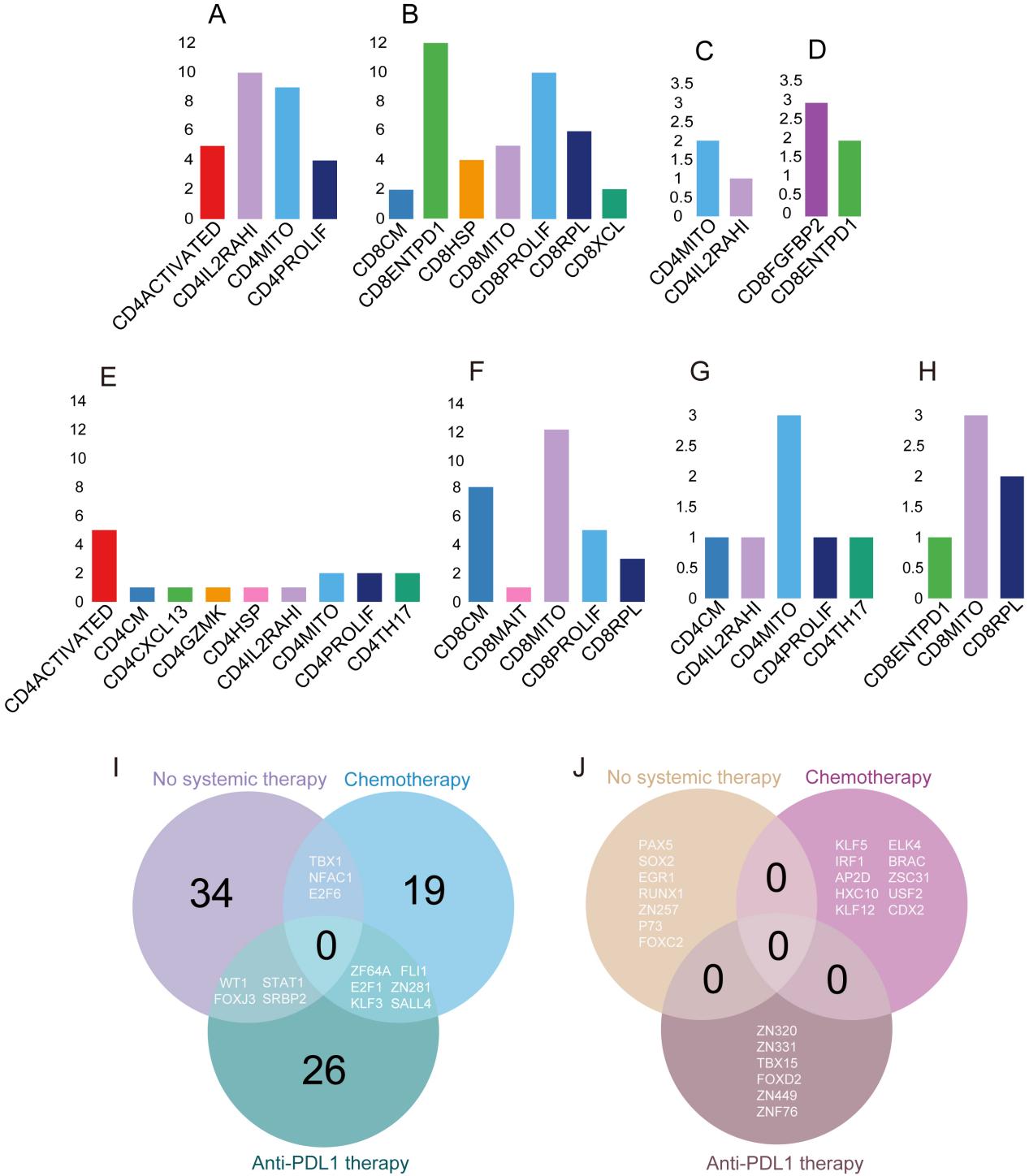


**Figure S1** CD4+/CD8+ T cell-specific transcription factors and their distribution among cell subtypes at the single-cell level across various treatment regimens for bladder cancer. **(A to D)** Frequency statistics of specific transcription factors for different cell subtypes in the no systemic therapy group. **A** and **B** represent the frequency of transcription factors in tumor samples under no systemic therapy, while **C** and **D** represent the frequency of transcription factors in normal samples under no systemic therapy. **(E to H)** Frequency statistics of specific transcription factors for different cell subtypes in the chemotherapy group. **E** and **F** represent the frequency of transcription factors in tumor samples after chemotherapy, while **G** and **H** represent the frequency of transcription factors in normal samples post-chemotherapy control treatment. The horizontal coordinates indicate the different cell subtypes and the vertical coordinates indicate the frequency of specific transcription factors. **(I)** indicates transcription factors specific and common to bladder cancer samples in different treatment regimens. **(J)** indicates transcription factors specific and common to normal tissue samples in different treatment regimens.

**3.2 Treatment Effects on Overall Interactions among CD8+ and CD4+ T Cell Subtypes**

Using Cellchat (*28*), we analyzed the scRNA-seq dataset to assess the intensity and volume of cellular communication within and between these cell subtypes under various treatment conditions. Our findings indicated that CD8+ T cells exhibited a significantly higher number and intensity of interactions compared to CD4+ T cells across all conditions (**Fig. 1 D-G, Fig. S2**). Specifically, chemotherapy was associated with a 2.4-fold increase in the number of interactions among CD8+ T cells compared to CD4+ T cells, and a 4.6-fold increase in interaction intensity (**Fig. S2** **E-H**). Furthermore, under anti-PD-L1 blockade, the interactions in CD8+ T cells escalated dramatically to 38,266 compared to 3,903 in CD4+ T cells, showcasing a stark disparity in communicative activity between the two cell types (**Fig. 1 D-G**).

Interestingly, both CD8+ and CD4+ T cells showed reduced interaction frequencies and intensities in the absence of systemic therapy, although CD8+ T cells remained notably more active than their CD4+ counterparts (**Fig. 1 D-G, Fig. S2**). This sustained activity of CD8+ T cells across treatment modalities underscores their pivotal role in the immune response against bladder cancer, potentially influencing the effectiveness of various therapeutic strategies. The study provides crucial insights into the dynamic cellular interactions within the tumor microenvironment, highlighting the influence of treatment regimens on immune cell behavior. This information is vital for optimizing treatment strategies and enhancing the overall efficacy of immunotherapy in bladder cancer.


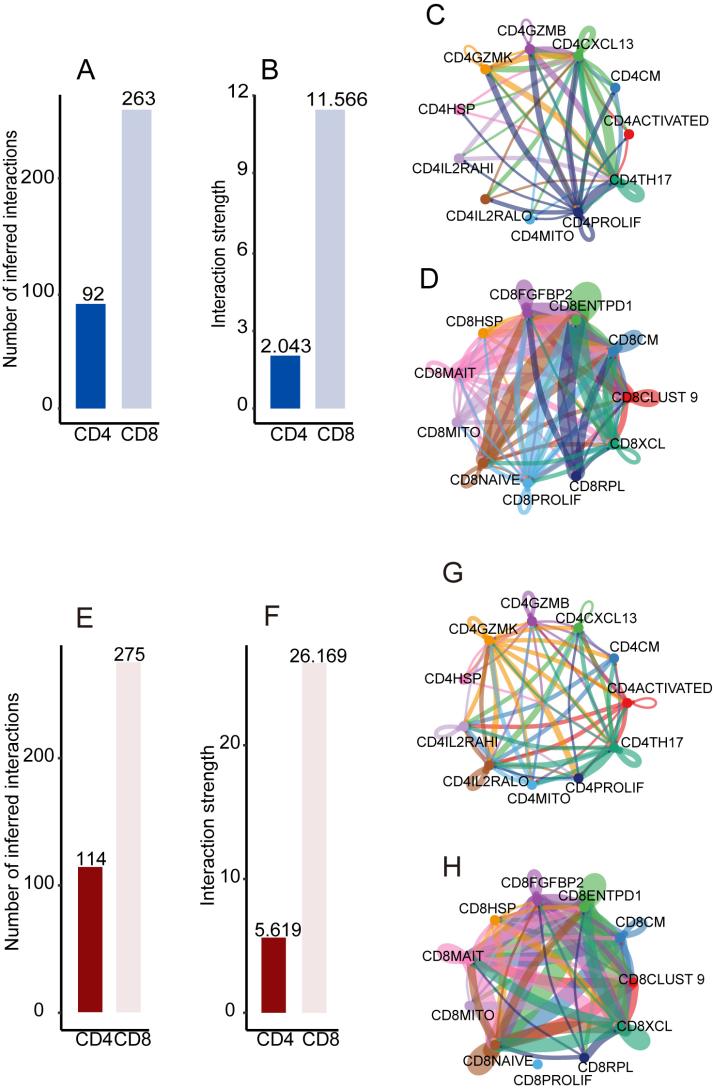


**Figure S2** Interaction intensity and number of T cells at the single cell level in different treatment regimens for bladder cancer. **(A to D)** Number of interactions, strength of interactions and interactions between different subtypes of T cells in the no systemic therapy group. **A** and **B** represent the number and intensity of interactions among CD4+/CD8+ T cells under no systemic therapy, while **C** and **D** depict the interaction network among CD4+/CD8+ T cells under no systemic therapy. Different colors represent different cell subtypes, and the thickness of the lines indicates the strength of the interactions. **(E to H)** Number of interactions, strength of interactions and interactions between different subtypes of T cells in the chemotherapy group. **E** and **F** represent the number and intensity of interactions among CD4+/CD8+ T cells under chemotherapy, while **G** and **H** depict the interaction network among CD4+/CD8+ T cells under chemotherapy. Different colors represent different cell subtypes, and the thickness of the lines indicates the strength of the interactions.

**3.3 Treatment Effects on Information Flow in Immune CD4+ T cells and CD8+ T cells**

The analysis of information flow between CD4+ T cells and CD8+ T cells, based on the summation of communication probabilities between cell populations, provides critical insights into how these cells respond to various treatment regimens (**Fig. 1 H and I, Fig. S3**). Our research identified several consistently activated pathways across different treatments, including CLEC (C-type lectin domain family 1 member B), MIF (macrophage migration inhibitory factor), CD99 (T-cell surface glycoprotein E2), LCK (T cell-specific protein-tyrosine kinase), and MHC-I (major histocompatibility complex, class I). Particularly noteworthy was the effect of anti-PD-L1 therapy, which led to the inactivation of pathways such as ITGB2 (integrin subunit beta 2), CCL (C-C motif chemokine ligand), CD70 (surface antigen CD70), and SPP1 (secreted phosphoprotein 1), distinguishing it from other treatment approaches. Furthermore, the consistent overlap in the activation of the MIF and CD99 pathways between CD4+ and CD8+ T cells under different treatment conditions highlights their critical role in modulating immune cell responses. This finding is crucial for understanding the mechanisms through which these pathways may enhance the efficacy of immunotherapeutic interventions, potentially leading to improved treatment outcomes.


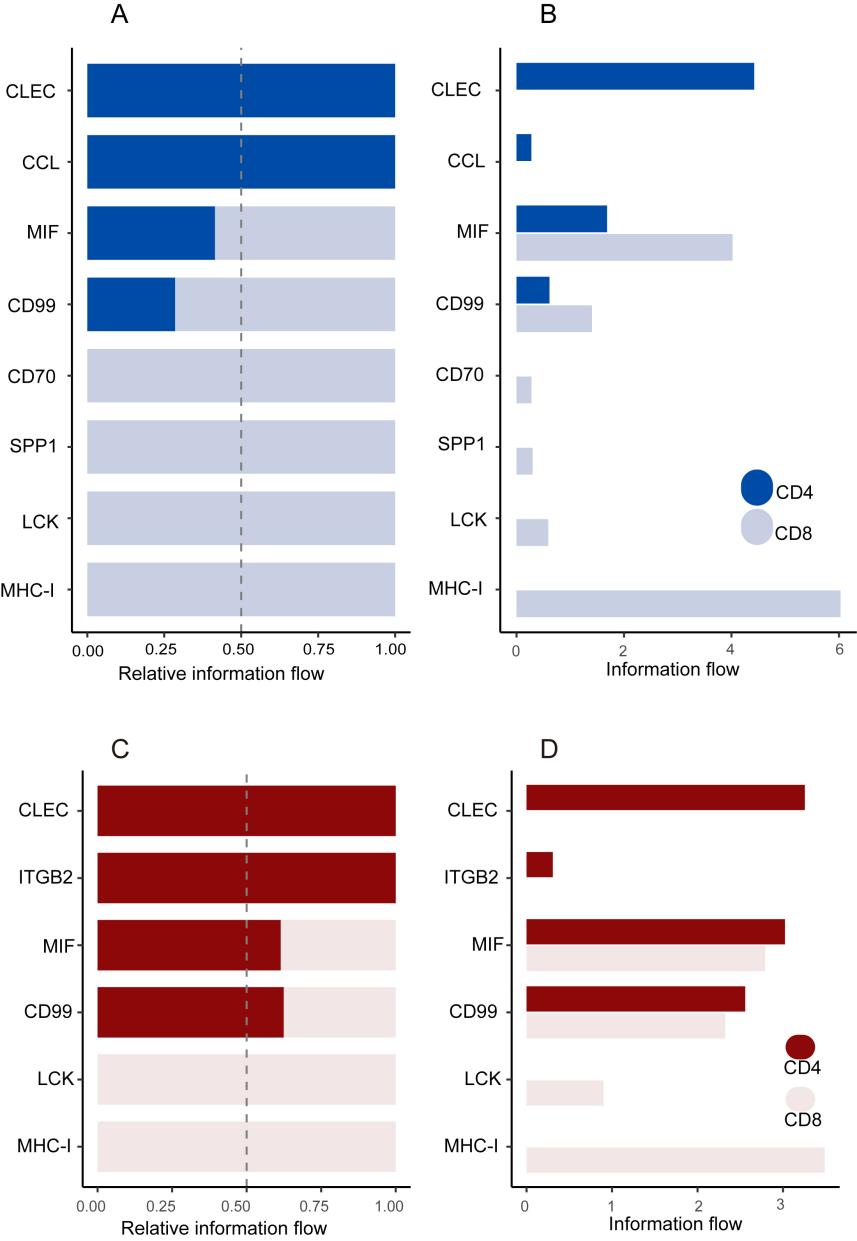


**Figure S3** Information flow of different subtypes of CD4+/CD8+ T cells at the single cell level in different treatment regimens for bladder cancer. **(A and B)** Incoming-outgoing interaction correlated signaling pathways and their relative proportions in the no systemic therapy group. **(C and D)** Incoming-outgoing interaction correlated signaling pathways and their relative proportions in the chemotherapy group. The vertical coordinates are abbreviated for different signaling pathways; the horizontal coordinates of A and C are relative information flow, and the horizontal coordinates of B and D are information flow.

**3.4 Communication Behavior Patterns of Bladder Cancer CD8+ and CD4+ T Cells**

We examined the input and output signaling patterns of T cell communication to differentiate among various T cell subtypes. Immune cell subtypes demonstrated distinct signaling patterns across different treatment regimens, with similarities and differences in communication among CD8+ T cells and CD4+ T cells revealed (**Fig. 1 J and K, Fig. S4**). Notably, certain immune cell subtypes were identified as inert, specifically CD4MITO, CD8MITO, CD8HSP, and CD8RPL T cells. Interestingly, some typically less active subtypes, such as CD4CM and CD8PROLIF T cells, became activated following anti-PD-L1 treatment, contrasting with their behavior under chemotherapy and without systemic therapy (**Fig. 1 J and K**).

The response to anti-PD-L1 treatment brought about notable shifts in signal intensities for many immune subtypes. In particular, CD8CLUST9 and CD8PROLIF T cells transitioned from predominantly output to balanced input-output signaling. Similarly, CD4PROLIF T cells showed alterations in signaling intensity, with both input and output signals being weaker in the anti-PD-L1 treatment group compared to the chemotherapy and no systemic therapy groups. Additionally, CD4CM, CD4HSP, CD4IL2RAHI, CD4IL2RALO, and CD4CXCL13 T cells displayed enhanced output signaling specifically in the anti-PD-L1 treatment group, indicating a dynamic adjustment in their communicative roles depending on the therapeutic environment (**Fig. 1 J and K, Fig. S4**). This detailed analysis highlights the adaptive changes in cellular communication within the tumor microenvironment, underscoring the complexity and specificity of immune responses under different therapeutic conditions.


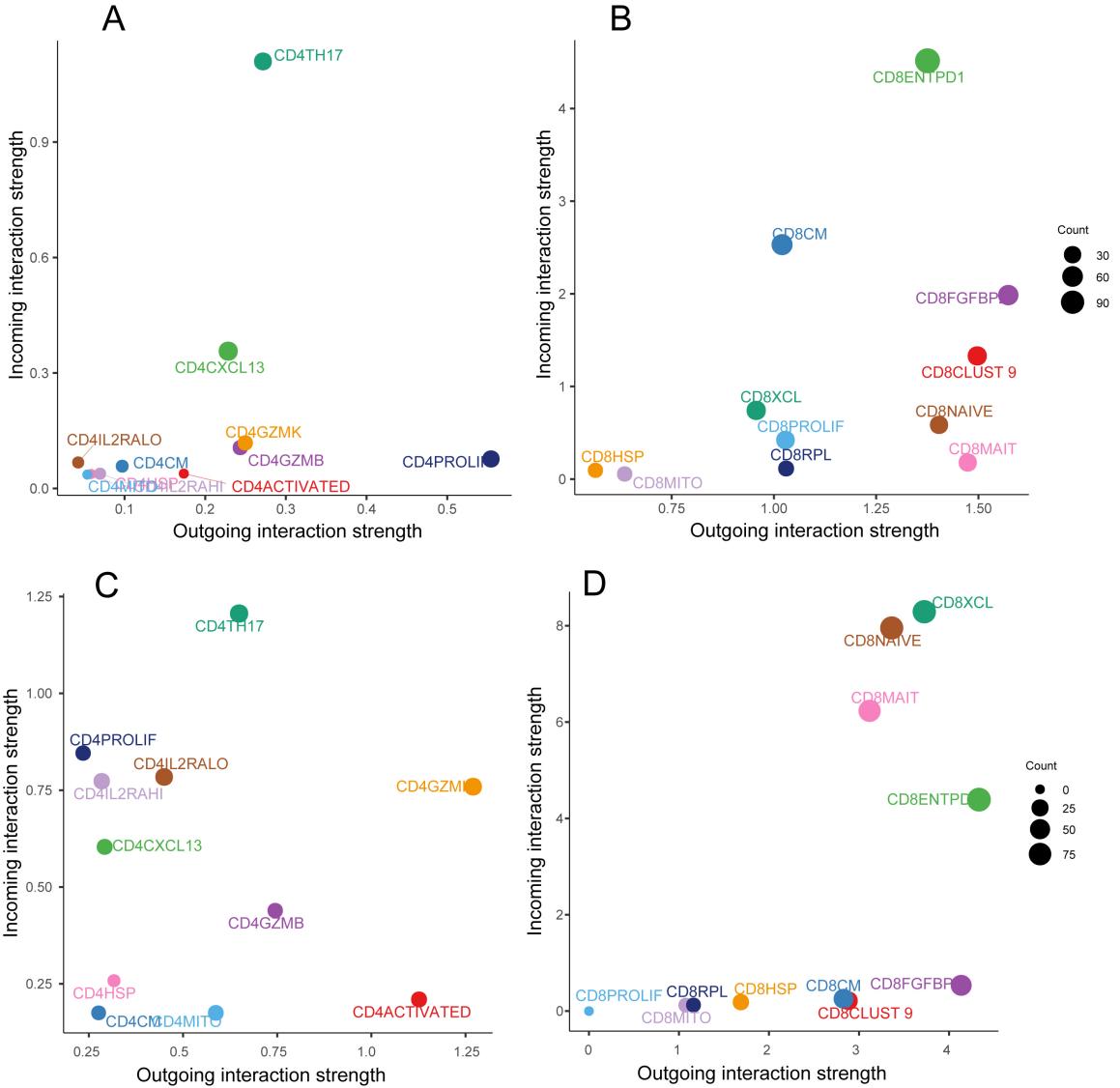
**Figure S4** Incoming-outgoing interaction intensity distribution of different subtypes of CD4+/CD8+ T cells at the single-cell level in different treatment regimens for bladder cancer. **(A and B)** The intensity of Incoming-outgoing interactions in the no systemic therapy group. **(C and D)** The intensity of Incoming-outgoing interactions in the chemotherapy group. The horizontal coordinate represents the outgoing interaction intensity and the vertical coordinate represents the incoming interaction intensity. The different colors of the circles represent different T cell subtypes, and the cell subtypes to which the circles belong are marked with the corresponding colors. The circle size indicates the count size of count.

**3.5 Involvement of T Cell Subtypes in Communication Patterns Across Treatment Regimens**

Across various treatment regimens, CD4+ and CD8+ T cells exhibited distinct involvement in the cellular communication of different subtypes, demonstrating variable engagement in immune pathways (**Fig. S5**). Notably, CD8+ T cells showed enhanced activity across all treatment conditions, engaging a broader array of cell types and pathways. This heightened activity has significant implications for refining and customizing future treatment protocols.


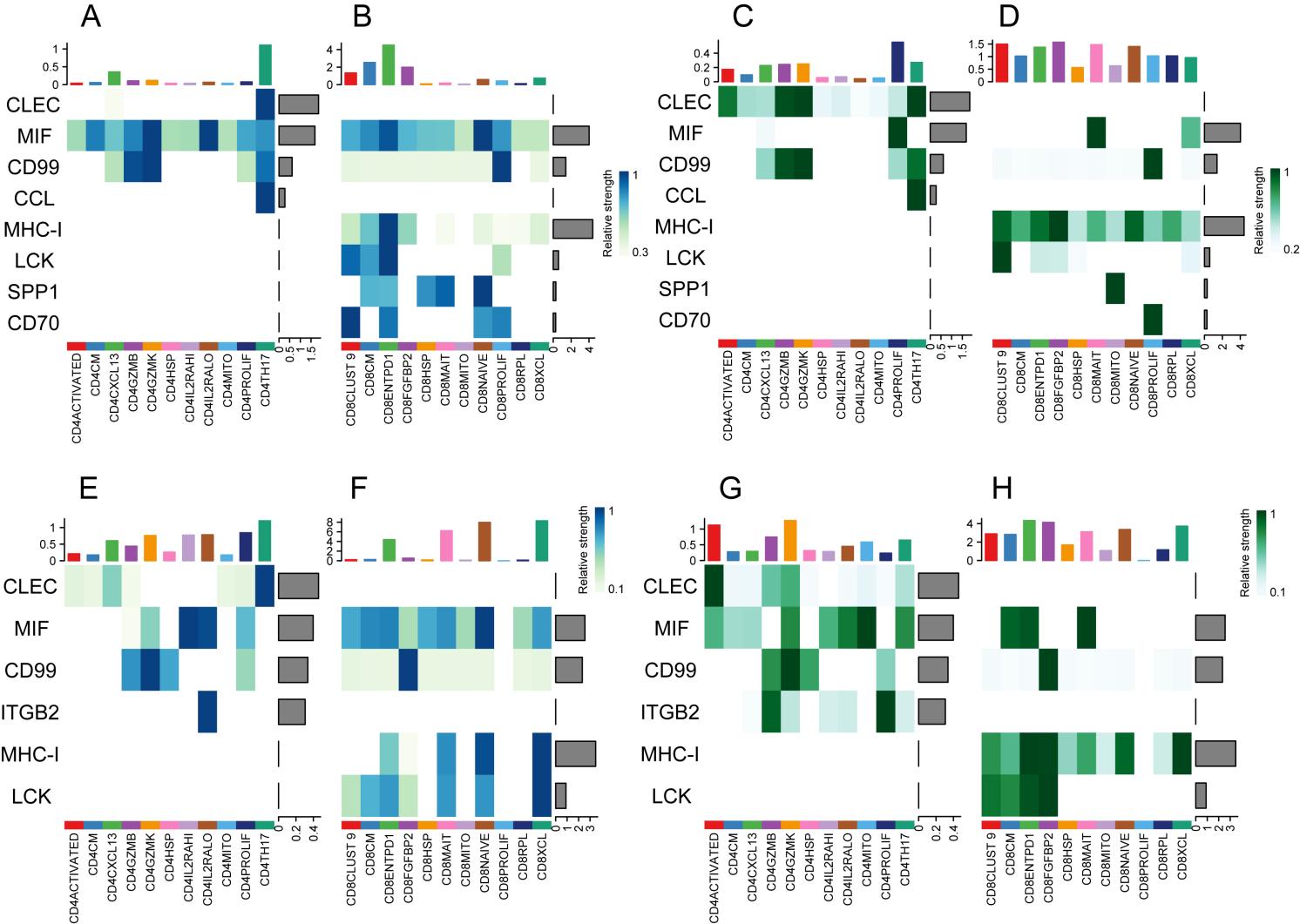


**Figure S5** Distinct signaling pathways of different CD4+/CD8+ T subtypes at the single cell level in different treatment regimens for bladder cancer. **(A to D)** Distribution of incoming-outgoing interaction correlated signaling pathways and their cell subtypes in the no systemic therapy group. **A** represents the incoming signaling patterns of CD4+ T cells under no systemic therapy, while **B** represents the incoming signaling patterns of CD8+ T cells under no systemic therapy. **C** indicates the outgoing signaling patterns of CD4+ T cells during no systemic therapy, and **D** indicates the outgoing signaling patterns of CD8+ T cells during no systemic therapy. **(E to H)** Distribution of incoming-outgoing interaction correlated signaling pathways and their cell subtypes in the chemotherapy group. **E** represents the incoming signaling patterns of CD4+ T cells under chemotherapy, while **F** represents the incoming signaling patterns of CD8+ T cells under chemotherapy. **G** indicates the outgoing signaling patterns of CD4+ T cells during chemotherapy, and **H** indicates the outgoing signaling patterns of CD8+ T cells during chemotherapy. The vertical coordinates of the heat map indicate the signaling pathways and the horizontal coordinates indicate the different cell subtypes. Heatmap colors indicate the relative strength of different pathways in different subtypes of cells. The top of the heat map indicates the relative strengths accounted for by different subtypes of cells, and the right side of the heat map indicates the strengths accounted for by different pathways.

During anti-PD-L1 treatment, there was an observable increase in the diversity of T cell subtypes involved in the overlapping CD99 and MIF pathways, suggesting that anti-PD-L1 therapy significantly expands the scope of cellular interactions, potentially enhancing immunotherapeutic efficacy. Conversely, in the CLEC pathway, CD8+ T cells activated a complete set of subtypes involved in output signaling, marking a distinct contrast to their behavior under chemotherapy conditions (**Fig. 1 L-O**).

In scenarios lacking systemic therapy, only a limited array of pathways was activated by CD4+ T cells, specifically CLEC, MIF, and CD99. Both CD4+ and CD8+ T cells displayed reduced activity compared to treatment conditions, involving fewer and less robustly engaged cell subtypes and pathways (**Fig. S5 A-D**). This differential engagement emphasizes the importance of understanding the specific cellular dynamics in untreated settings, which could guide the development of less invasive management strategies for certain patient populations.

In chemotherapy settings, specific CD4+ T cell subtypes, such as CD4GZMB and CD4GZMK, were predominantly involved in pathways overlapping with CD8+ T cells. In contrast, within the CD99 pathway, CD8FGFBP2 T cells were the primary correspondents among CD8+ T cells. Moreover, except for CD8PROLIF T cells, all subtypes of CD8+ T cells participated in the MIF pathway, illustrating a comprehensive engagement in this crucial immunomodulatory route (**Fig. S5 E-H**).

**3.6 The active behavior of CD8 in immunotherapy is supported by active ligand-receptor pairs**

In our analysis of the frequency of active transcription factors and cellular communication behaviors, we observed that CD8+ T cells exhibited relatively more active cellular behavior compared to CD4+ T cells. Further substantiation through analysis of their ligand-receptor pairs also supported this observation. We observed that samples with no systemic therapy were relatively inactive compared to those undergoing chemotherapy and immunotherapy, exhibiting limited activity in only a few ligand-receptor pairs, such as HLA-C and CD8A (**Fig. S6**). In the chemotherapy and immunotherapy groups, we observed that the Major Histocompatibility Complex, Class I series and the T-Cell Surface Glycoprotein CD8 series of receptors predominated (*47*). Recent research has identified NKG2A/CD94 as a novel immune checkpoint target, reflecting a growing interest in expanding the repertoire of therapeutic targets in immunotherapy. Interestingly, our findings indicated that HLA-E paired with CD94:NKG2A receptors were prevalent in the untreated group, whereas HLA-E and CD94:NKG2C receptors were detected in the group treated with immunotherapy. These receptors are part of the Killer Cell Lectin-Like Receptor series, and while they are not predominant, their differential expression before and after immunotherapy offers valuable insights into the mechanisms of immune checkpoint inhibition, suggesting potential avenues for therapeutic intervention (*48, 49*).

**
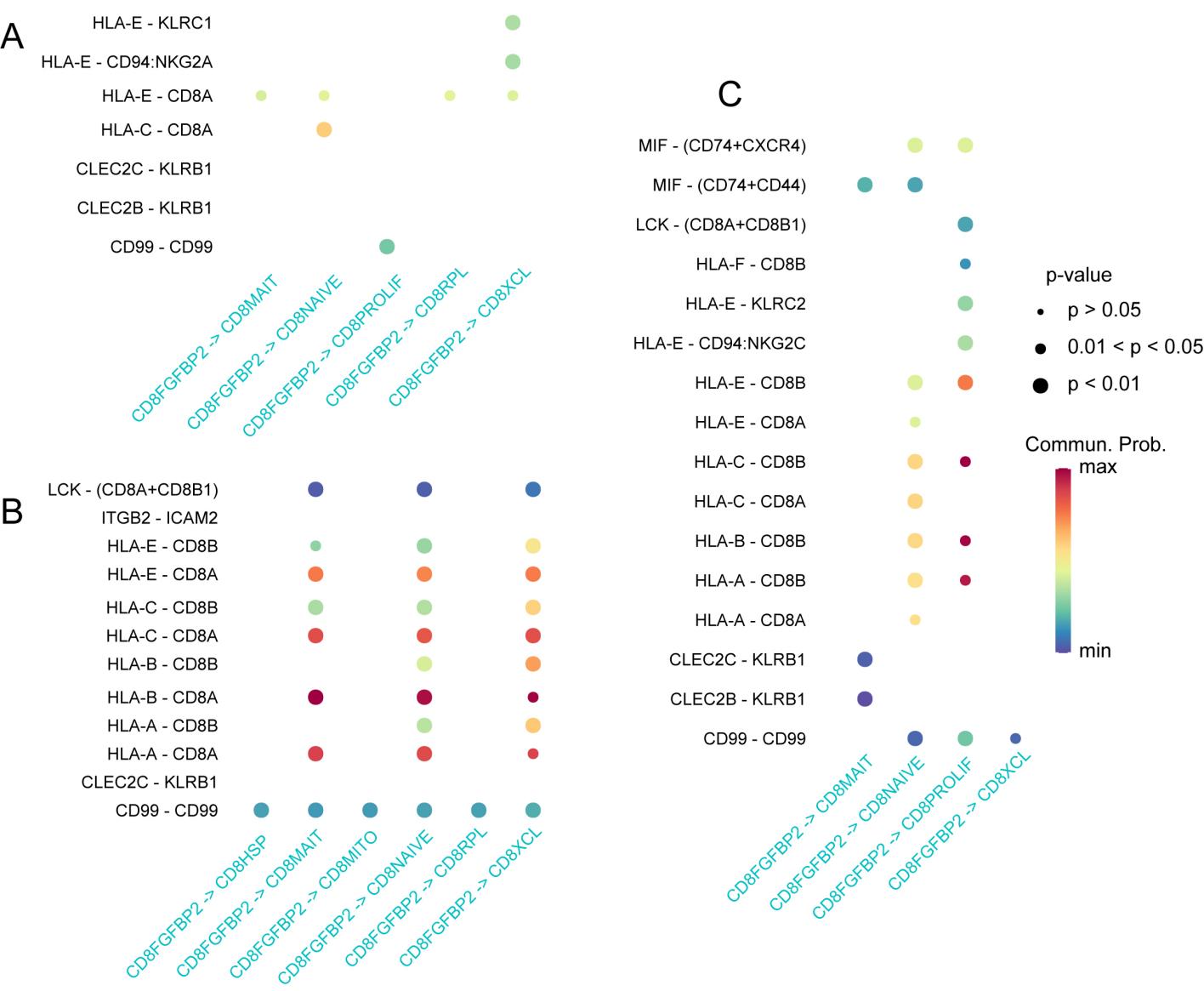
**

**Figure S6** Active states of T cell ligand-receptor pairs under different treatment conditions. **A** represents the active ligand-receptor status of T cells with no systemic treatment, **B** represents the active ligand-receptor status of T cells under chemotherapy, and **C** represents the active ligand-receptor status of T cells under Anti-PD-L1 treatment.

**4.Discussion**

Immunotherapy exhibits limited effectiveness in treating solid tumors, with only about 20% of such cases responding to treatment (*17, 18*). To refine immunotherapy regimens, it is essential to investigate the behavioral patterns and molecular characteristics of different cell subtypes in solid tumors like bladder cancer, both before and after treatment. This involves understanding how different subtypes collaborate, identifying similarities between CD8+ and CD4+ T cells, and exploring common molecular patterns. Our study dives deep into these aspects at the single-cell level, examining T cell interactions and transcriptional regulatory networks across various treatment regimens. Notably, this research provides the first quantitative analysis of intercellular communication among bladder cancer immune cells using social network analysis, pattern recognition, and manifold learning, identifying significant roles and common features within T cell subpopulations (*28*). This is also the first study to analyze regulons across different CD4+ and CD8+ T cell subtypes in bladder cancer under varying treatment conditions, pinpointing transcription factors unique to specific treatments.

**4.1 Coordinated CD4+ T cell and CD8+ T cell functions contribute to immunotherapy**

Our key finding was the observation that coordination of CD4+ T cell and CD8+ T cell functions contributes to immunotherapy. The expansion and development of tumor-infiltrating immune cells is a process of dynamic balance associated with many factors in the microenvironment, such as chemotherapeutic agents and immunotherapeutic agents. However, previous studies ignored the fact that these immune T cells require specific patterns of communication and transfer during migration and expansion. To the best of our knowledge, this is the first study to investigate signaling exchanges among T cell subsets in bladder cancer patients across different treatment regimens, and likely the first to compare the communication patterns of T cells in different treatment regimens.

The relative contribution of cytotoxic T cells to tumor immunotherapy has been a key question that has remained unanswered (*14*). In early preclinical studies, CD8-mediated immunity was found to be impaired and cytotoxic CD4+ T cells could be expanded to provide immune protection (*50-52*). Our first attempt to analyze cell behavior based on a social network and pattern recognition approach identified that some subtypes of CD4+ T cells and CD8+ T cells were similar in their input and output interaction intensity behavior and that they may work in concert with each other to kill tumor cells. CD4_GZMK_ and CD4_GZMB_ CD4+ T cell behaviors were similar to those of the CD8+ T cell subtypes CD8_CLUST9_, CD8_PROLIF_, CD8_CM_, and CD8_NAIVE_ T cells. Even though there was a greater similarity in the intensity of cellular input and output in the different treatment regimens, there was always an overlap within the pathways activated by input and output. CD4_GZMK_ and CD4_GZMB_ T cells expressing cytolytic granzymes and perforin for tumor cell killing may work in concert with CD8_CLUST9_, CD8_PROLIF_, CD8_CM_, and CD8_NAIVE_ T cells to carry out clearance of tumor cells. Conversely, there were cellular subtypes with non-overlapping cell behavior such as CD4_TH17_ and CD8_ENTPD1_ T cells, which did not have overlapping pathways between them. That is, CD4_TH17_ T cells were involved in a pathway largely uninvolved with CD8_ENTPD1_ T cells, and conversely, CD8_ENTPD1_ T cells were involved in a pathway largely uninvolved with CD4_TH17_ T cells. The pivotal cells in the communication pattern, CD4_TH17_ and CD8_ENTPD1_ T cells, are the mainstays of immune cells in the tumor microenvironment. Consistent with previous studies, the CD4_TH17_ and CD8_ENTPD1_ T cells were the more lethal cell subpopulations (*53-57*), and they both had more active signal output intensities, which is one of the common molecular features across the different treatment regimens. Both types of cells had different active communication pathways, suggesting that they performed different tasks in the tumor microenvironment to counter the expansion of tumor cells. These cell subtypes with similar behavior or intensity can be used as important reference indicators during immunotherapy, such as their similar behavior and intensity of contributions between immune cell states of different patients, which can provide a reference for the intensity of different cell behavioral activities and further rapid selection of more targeted immune cell subtypes, which is important for individualized medicine.

**4.2 Common molecular patterns of CD4+ T cells and CD8+ T cells may affect treatment outcome**

Common molecular patterns of CD4+ and CD8+ T cells were evident in our study. Many of the specific transcription factors involved with cytotoxic CD4+ T cells were similar to specific transcription factors of CD8+ T cells, with the main common transcription factors the KLF family, WT1, and NFAC1, all of which are linked to T cell functions such as receptor activation. Additional preclinical evidence is needed on whether CD8+/CD4+ T cell can be combined with drugs that target the KLF family (e.g., simvastatin, cisplatin, and morphine) (*58-61*). In recent years, WT1-expressing solid tumor cell lines, primary acute myeloid leukemia (AML) blasts, and ovarian cancer patient samples were found to be antitumor responsive, particularly those with WT1-specific T cell receptors that improved antitumor T cell function (*40, 62, 63*). Deletion of NFAC1 affects T cell activation and functional limitation, favoring tumor cell growth (*64*).

However, we also observed many different transcription factors specific to cytotoxic CD4+ T cells compared to the transcription factors specific to some of the CD8+ T cells. This suggests that the production and functioning of cytotoxic CD4+ T cells and CD8+ T cells may be a complex and ongoing dynamic evolutionary process with a large cellular and molecular signature in the different therapeutic microenvironments of bladder cancer patient heterogeneity.

**5.Conclusions**

Our study elucidates the complex interactions and molecular similarities between CD4+ and CD8+ T cells within solid tumors, significantly advancing the potential for future immunotherapy strategies. However, it is crucial to note the limitations concerning the spatial distribution of immune cell subtypes, which were not fully captured in this study (*65-69*). Future applications involving spatial transcriptome and proteomic technologies are expected to significantly enhance our understanding of the spatial dynamics of immune cells in bladder cancer, potentially revolutionizing the precision and efficacy of immunotherapy (*70, 71*).

**Acknowledgement**

Thanks to PhD J.J.W., Professor K.L. and Professor C.C. for providing the research space and server.

**Author contributions**

J.J.W. designed the study. J.J.W. and X.W. wrote the manuscript. J.J.W. and S.D.L. were responsible for editing the images and processing the single-cell data. J.J.W., X.W., S.L., C.J.Y., H.M.X., J.Y.W., L.Z., M.L., R.C., S.D.L., P.W., J.Z.D., Y.L., L.Z., K.D., H.Y.H., and T.W.L. participated in the discussion section of the article. J.J.W., C.C., L.M.L., X.W., K.L., and J.Y.W. for an in-depth discussion of the clinical value of immunotherapy.

**Declaration of Interests Statement**

The authors declare that there are no conflicts of interest associated with this work.

**Supplementary Table**

**Supplementary Table 1** Differentially expressed genes between different subtypes of CD4+ T cells and CD8+ T cells

**Supplementary Table 2** Regulons and their target genes in the no systemic therapy groups of different CD4+ T cell subtypes in normal bladder samples

**Supplementary Table 3** Regulons and their target genes in the no systemic therapy groups of different CD8+ T cell subtypes in normal bladder samples

**Supplementary Table 4** Regulons and their target genes in the no systemic therapy groups of different CD4+ T cell subtypes in bladder cancer samples

**Supplementary Table 5** Regulons and their target genes in the no systemic therapy groups of different CD8+ T cell subtypes in bladder cancer samples

**Supplementary Table 6** Regulons and their target genes in chemotherapy groups of different CD4+ T cell subtypes in normal bladder samples

**Supplementary Table 7** Regulons and their target genes in chemotherapy groups of different CD8+ T cell subtypes in normal bladder samples

**Supplementary Table 8** Regulons and their target genes in chemotherapy groups of different CD4+ T cell subtypes in bladder cancer samples

**Supplementary Table 9** Regulons and their target genes in chemotherapy groups of different CD8+ T cell subtypes in bladder cancer samples

**Supplementary Table 10** Regulons and their target genes in anti-PDL1 groups of different CD4+ T cell subtypes in normal bladder samples

**Supplementary Table 11** Regulons and their target genes in anti-PDL1 groups of different CD4+ T cell subtypes in bladder cancer samples

**Supplementary Table 12** Regulons and their target genes in anti-PDL1 groups of different CD8+ T cell subtypes in bladder cancer samples

**Supplementary Table 13** Distribution of immune cell-specific transcription factors and their cell types in different treatment regimens for bladder cancer

**References**

1. G. Morad, B. A. Helmink, P. Sharma, J. A. Wargo, Hallmarks of response, resistance, and toxicity to immune checkpoint blockade. *Cell* **184**, 5309-5337 (2021).

2. G. L. Ma, W. F. Lin, Immune checkpoint inhibition mediated with liposomal nanomedicine for cancer therapy. *Military Medical Research* **10**, 20 (2023).

3. M. C. Sellars, C. J. Wu, E. F. Fritsch, Cancer vaccines: Building a bridge over troubled waters. *Cell* **185**, 2770-2788 (2022).

4. F. Baharom *et al.*, Systemic vaccination induces CD8(+) T cells and remodels the tumor microenvironment. *Cell* **185**, 4317-4332 e4315 (2022).

5. G. M. Lynn *et al.*, Peptide-TLR-7/8a conjugate vaccines chemically programmed for nanoparticle self-assembly enhance CD8 T-cell immunity to tumor antigens. *Nature biotechnology* **38**, 320-332 (2020).

6. F. Baharom *et al.*, Intravenous nanoparticle vaccination generates stem-like TCF1(+) neoantigen-specific CD8(+) T cells. *Nature immunology* **22**, 41-52 (2021).

7. A. M. Luoma *et al.*, Tissue-resident memory and circulating T cells are early responders to pre-surgical cancer immunotherapy. *Cell* **185**, 2918-2935 e2929 (2022).

8. A. Chow, K. Perica, C. A. Klebanoff, J. D. Wolchok, Clinical implications of T cell exhaustion for cancer immunotherapy. *Nature reviews. Clinical oncology* **19**, 775-790 (2022).

9. L. Labanieh *et al.*, Enhanced safety and efficacy of protease-regulated CAR-T cell receptors. *Cell* **185**, 1745-1763 e1722 (2022).

10. K. Pan *et al.*, CAR race to cancer immunotherapy: from CAR T, CAR NK to CAR macrophage therapy. *Journal of experimental & clinical cancer research : CR* **41**, 119 (2022).

11. H. Sung *et al.*, Global Cancer Statistics 2020: GLOBOCAN Estimates of Incidence and Mortality Worldwide for 36 Cancers in 185 Countries. *CA: a cancer journal for clinicians* **71**, 209-249 (2021).

12. J. Afonso, L. L. Santos, A. Longatto-Filho, F. Baltazar, Competitive glucose metabolism as a target to boost bladder cancer immunotherapy. *Nature reviews. Urology* **17**, 77-106 (2020).

13. D. Y. Oh *et al.*, Intratumoral CD4(+) T Cells Mediate Anti-tumor Cytotoxicity in Human Bladder Cancer. *Cell* **181**, 1612-1625 e1613 (2020).

14. D. Y. Oh, L. Fong, Cytotoxic CD4(+) T cells in cancer: Expanding the immune effector toolbox. *Immunity* **54**, 2701-2711 (2021).

15. C. Kandoth *et al.*, Mutational landscape and significance across 12 major cancer types. *Nature* **502**, 333-339 (2013).

16. A. D. Waldman, J. M. Fritz, M. J. Lenardo, A guide to cancer immunotherapy: from T cell basic science to clinical practice. *Nat Rev Immunol* **20**, 651-668 (2020).

17. K. M. Hargadon, C. E. Johnson, C. J. Williams, Immune checkpoint blockade therapy for cancer: An overview of FDA-approved immune checkpoint inhibitors. *International immunopharmacology* **62**, 29-39 (2018).

18. S. Mariathasan *et al.*, TGFbeta attenuates tumour response to PD-L1 blockade by contributing to exclusion of T cells. *Nature* **554**, 544-548 (2018).

19. C. C. Balanca *et al.*, PD-1 blockade restores helper activity of tumor-infiltrating, exhausted PD-1hiCD39+ CD4 T cells. *JCI insight* **6**, (2021).

20. A. M. van der Leun, D. S. Thommen, T. N. Schumacher, CD8(+) T cell states in human cancer: insights from single-cell analysis. *Nature reviews. Cancer* **20**, 218-232 (2020).

21. L. Zheng *et al.*, Pan-cancer single-cell landscape of tumor-infiltrating T cells. *Science (New York, N.Y.)* **374**, abe6474 (2021).

22. C. Zheng *et al.*, Landscape of Infiltrating T Cells in Liver Cancer Revealed by Single-Cell Sequencing. *Cell* **169**, 1342-1356 e1316 (2017).

23. X. Guo *et al.*, Global characterization of T cells in non-small-cell lung cancer by single-cell sequencing. *Nature medicine* **24**, 978-985 (2018).

24. L. Zhang *et al.*, Lineage tracking reveals dynamic relationships of T cells in colorectal cancer. *Nature* **564**, 268-272 (2018).

25. P. Savas *et al.*, Single-cell profiling of breast cancer T cells reveals a tissue-resident memory subset associated with improved prognosis. *Nature medicine* **24**, 986-993 (2018).

26. H. Raskov, A. Orhan, J. P. Christensen, I. Gogenur, Cytotoxic CD8(+) T cells in cancer and cancer immunotherapy. *British journal of cancer* **124**, 359-367 (2021).

27. J. Galon, D. Bruni, Approaches to treat immune hot, altered and cold tumours with combination immunotherapies. *Nature reviews. Drug discovery* **18**, 197-218 (2019).

28. S. Jin *et al.*, Inference and analysis of cell-cell communication using CellChat. *Nat Commun* **12**, 1088 (2021).

29. A. Ma *et al.*, IRIS3: integrated cell-type-specific regulon inference server from single-cell RNA-Seq. *Nucleic Acids Res* **48**, W275-W286 (2020).

30. T. L. Bailey *et al.*, MEME SUITE: tools for motif discovery and searching. *Nucleic Acids Res* **37**, W202-208 (2009).

31. J. Yang, X. Chen, A. McDermaid, Q. Ma, DMINDA 2.0: integrated and systematic views of regulatory DNA motif identification and analyses. *Bioinformatics (Oxford, England)* **33**, 2586-2588 (2017).

32. J. Xie *et al.*, QUBIC2: a novel and robust biclustering algorithm for analyses and interpretation of large-scale RNA-Seq data. *Bioinformatics (Oxford, England)* **36**, 1143-1149 (2020).

33. X. Qiu *et al.*, Single-cell mRNA quantification and differential analysis with Census. *Nature methods* **14**, 309-315 (2017).

34. W. Gong, I. Y. Kwak, P. Pota, N. Koyano-Nakagawa, D. J. Garry, DrImpute: imputing dropout events in single cell RNA sequencing data. *BMC bioinformatics* **19**, 220 (2018).

35. K. Street *et al.*, Slingshot: cell lineage and pseudotime inference for single-cell transcriptomics. *BMC genomics* **19**, 477 (2018).

36. A. T. Lun, D. J. McCarthy, J. C. Marioni, A step-by-step workflow for low-level analysis of single-cell RNA-seq data with Bioconductor. *F1000Res* **5**, 2122 (2016).

37. T. L. Bailey, J. Johnson, C. E. Grant, W. S. Noble, The MEME Suite. *Nucleic Acids Res* **43**, W39-49 (2015).

38. N. Wang *et al.*, TBX1 Functions as a Tumor Suppressor in Thyroid Cancer Through Inhibiting the Activities of the PI3K/AKT and MAPK/ERK Pathways. *Thyroid : official journal of the American Thyroid Association* **29**, 378-394 (2019).

39. Y. Liu *et al.*, A positive feedback loop of CENPU/E2F6/E2F1 facilitates proliferation and metastasis via ubiquitination of E2F6 in hepatocellular carcinoma. *International journal of biological sciences* **18**, 4071-4087 (2022).

40. E. Ruggiero *et al.*, CRISPR-based gene disruption and integration of high-avidity, WT1-specific T cell receptors improve antitumor T cell function. *Science translational medicine* **14**, eabg8027 (2022).

41. Y. Verhoeven *et al.*, The potential and controversy of targeting STAT family members in cancer. *Seminars in cancer biology* **60**, 41-56 (2020).

42. Y. J. Li, C. Zhang, A. Martincuks, A. Herrmann, H. Yu, STAT proteins in cancer: orchestration of metabolism. *Nature reviews. Cancer* **23**, 115-134 (2023).

43. G. Barisciano *et al.*, The miR-27a/FOXJ3 Axis Dysregulates Mitochondrial Homeostasis in Colorectal Cancer Cells. *Cancers* **13**, (2021).

44. Z. Chen *et al.*, In vivo CD8(+) T cell CRISPR screening reveals control by Fli1 in infection and cancer. *Cell* **184**, 1262-1280.e1222 (2021).

45. F. González-Romero *et al.*, E2F1 and E2F2-Mediated Repression of CPT2 Establishes a Lipid-Rich Tumor-Promoting Environment. *Cancer research* **81**, 2874-2887 (2021).

46. Z. Huang, H. He, F. Qiu, H. Qian, Expression and Prognosis Value of the KLF Family Members in Colorectal Cancer. *Journal of oncology* **2022**, 6571272 (2022).

47. C. Battin *et al.*, NKG2A-checkpoint inhibition and its blockade critically depends on peptides presented by its ligand HLA-E. *Immunology* **166**, 507-521 (2022).

48. S. P. Patel *et al.*, Phase 1/2 study of monalizumab plus durvalumab in patients with advanced solid tumors. *Journal for immunotherapy of cancer* **12**, (2024).

49. J. Middelburg *et al.*, The MHC-E peptide ligands for checkpoint CD94/NKG2A are governed by inflammatory signals, whereas LILRB1/2 receptors are peptide indifferent. *Cell Rep* **42**, 113516 (2023).

50. Y. Xie *et al.*, Naive tumor-specific CD4(+) T cells differentiated in vivo eradicate established melanoma. *The Journal of experimental medicine* **207**, 651-667 (2010).

51. S. A. Quezada *et al.*, Tumor-reactive CD4(+) T cells develop cytotoxic activity and eradicate large established melanoma after transfer into lymphopenic hosts. *The Journal of experimental medicine* **207**, 637-650 (2010).

52. D. Hirschhorn-Cymerman *et al.*, Induction of tumoricidal function in CD4+ T cells is associated with concomitant memory and terminally differentiated phenotype. *The Journal of experimental medicine* **209**, 2113-2126 (2012).

53. M. O. Johnson *et al.*, Distinct Regulation of Th17 and Th1 Cell Differentiation by Glutaminase-Dependent Metabolism. *Cell* **175**, 1780-1795 e1719 (2018).

54. S. Jiao *et al.*, Differences in Tumor Microenvironment Dictate T Helper Lineage Polarization and Response to Immune Checkpoint Therapy. *Cell* **179**, 1177-1190 e1113 (2019).

55. A. Basu *et al.*, Differentiation and Regulation of T(H) Cells: A Balancing Act for Cancer Immunotherapy. *Frontiers in immunology* **12**, 669474 (2021).

56. K. F. Corral-Jara, G. Rosas da Silva, N. A. Fierro, V. Soumelis, Modeling the Th17 and Tregs Paradigm: Implications for Cancer Immunotherapy. *Frontiers in cell and developmental biology* **9**, 675099 (2021).

57. M. Segovia, S. Russo, M. R. Girotti, G. A. Rabinovich, M. Hill, Role of inflammasome activation in tumor immunity triggered by immune checkpoint blockers. *Clinical and experimental immunology* **200**, 155-162 (2020).

58. L. Lu *et al.*, Kruppel-like factor 2 mediated anti-proliferative and anti-metastasis effects of simvastatin in p53 mutant colon cancer. *Biochemical and biophysical research communications* **511**, 772-779 (2019).

59. R. Li, J. Chen, X. Gao, G. Jiang, Transcription factor KLF2 enhances the sensitivity of breast cancer cells to cisplatin by suppressing kinase WEE1. *Cancer biology & therapy* **22**, 465-477 (2021).

60. S. Suzuki, L. F. Chuang, R. H. Doi, R. Y. Chuang, Identification of opioid-regulated genes in human lymphocytic cells by differential display: upregulation of Kruppel-like factor 7 by morphine. *Exp Cell Res* **291**, 340-351 (2003).

61. Z. Y. Li, Y. X. Zhu, J. R. Chen, X. Chang, Z. Z. Xie, The role of KLF transcription factor in the regulation of cancer progression. *Biomedicine & pharmacotherapy = Biomedecine & pharmacotherapie* **162**, 114661 (2023).

62. R. A. van Amerongen *et al.*, WT1-specific TCRs directed against newly identified peptides install antitumor reactivity against acute myeloid leukemia and ovarian carcinoma. *Journal for immunotherapy of cancer* **10**, (2022).

63. M. C. Lahman *et al.*, Targeting an alternate Wilms' tumor antigen 1 peptide bypasses immunoproteasome dependency. *Science translational medicine* **14**, eabg8070 (2022).

64. L. Heim *et al.*, NFATc1 Promotes Antitumoral Effector Functions and Memory CD8(+) T-cell Differentiation during Non-Small Cell Lung Cancer Development. *Cancer research* **78**, 3619-3633 (2018).

65. L. Tian, F. Chen, E. Z. Macosko, The expanding vistas of spatial transcriptomics. *Nature biotechnology* **41**, 773-782 (2023).

66. G. Palla, D. S. Fischer, A. Regev, F. J. Theis, Spatial components of molecular tissue biology. *Nature biotechnology* **40**, 308-318 (2022).

67. A. Rao, D. Barkley, G. S. Franca, I. Yanai, Exploring tissue architecture using spatial transcriptomics. *Nature* **596**, 211-220 (2021).

68. E. Lundberg, G. H. H. Borner, Spatial proteomics: a powerful discovery tool for cell biology. *Nature reviews. Molecular cell biology* **20**, 285-302 (2019).

69. A. Mund, A. D. Brunner, M. Mann, Unbiased spatial proteomics with single-cell resolution in tissues. *Molecular cell* **82**, 2335-2349 (2022).

70. L. Zhang *et al.*, Clinical and translational values of spatial transcriptomics. *Signal transduction and targeted therapy* **7**, 111 (2022).

71. K. Vandereyken, A. Sifrim, B. Thienpont, T. Voet, Methods and applications for single-cell and spatial multi-omics. *Nature reviews. Genetics* **24**, 494-515 (2023).
